# Quality of life in euthymic patients with bipolar disorder

**DOI:** 10.1192/j.eurpsy.2021.522

**Published:** 2021-08-13

**Authors:** N. Smaoui, I. Lajmi, A. Guermazi, M. Maalej Bouali, S. Omri, R. Feki, L. Zouari, N. Charfi, J. Ben Thabet, M. Maalej

**Affiliations:** 1 Psychiatry C Department, Hedi chaker university hospital, sfax, Tunisia; 2 Department Of Psychiatry “c”, Hedi Chaker University Hospital, Sfax, Tunisia

**Keywords:** bipolar disorder, quality of life, euthymic patients

## Abstract

**Introduction:**

Patients with bipolar disorder (BD) frequently experience residual symptoms, problems in psychosocial functioning, cognitive impairment, and poor quality of life (QOL).

**Objectives:**

* To evaluate the QOL of euthymic patients with BD compared to healthy controls (HC). * To identify factors associated with its deterioration.

**Methods:**

This is a comparative and analytical study, conducted over 3 months, involving 30 euthymic patients with BD, followed up in the outpatient psychiatry department of Hedi Chaker University Hospital in Sfax (Tunisia). They were compared to 34 HC. General, clinical and therapeutic data were collected using a pre-established questionnaire. QOL was assessed with the «36 item Short-Form Health Survey» (SF-36).

**Results:**

The mean ages of BD patients and HC were 44.17 years and 40.1 years, respectively. Compared with HC, patients with BD had decreased overall SF-36 scores (53.73 vs 73.78; p=0.000) and decreased physical and psychological subdomain scores (p=0.001; p=0.000). The study of the relationship between the dimensional average scores and different variables revealed correlations between; physical health problems and somatic disease (p=0.021) and unemployment (p=0.001), impaired general health and somatic disease (p=0.02) and psychotropic association (p=0.021), emotional health problems and psychiatric family history (p=0.023), pain and psychotropic association (p=0.031), and impaired global QOL and psychiatric family history (p=0.05).

**Conclusions:**

Our results confirm the impairment of the QOL of patients with BD even in euthymic periods. Many factors have been associated, including demographic and clinical variables. The improvement of QOL is to consider these factors in the management of these patients.

